# Multiple Single Nucleotide Polymorphism Testing Improves the Prediction of Diabetic Retinopathy Risk with Type 2 Diabetes Mellitus

**DOI:** 10.3390/jpm11080689

**Published:** 2021-07-21

**Authors:** Yu-Ting Hsiao, Feng-Chih Shen, Shao-Wen Weng, Pei-Wen Wang, Yung-Jen Chen, Jong-Jer Lee

**Affiliations:** 1 Department of Ophthalmology, Kaohsiung Chang Gung Memorial Hospital and Chang Gung University College of Medicine, Kaohsiung 83301, Taiwan; yuting1008@cgmh.org.tw (Y.-T.H.); f75622@cgmh.org.tw (Y.-J.C.); 2 Division of Metabolism and Endocrinology, Department of Internal Medicine, Kaohsiung Chang Gung Memorial Hospital and Chang Gung University College of Medicine, Kaohsiung 83301, Taiwan; carcinom@cgmh.org.tw (F.-C.S.); wengsw99@adm.cgmh.org.tw (S.-W.W.); wangpw@adm.cgmh.org.tw (P.-W.W.); 3 Center for Mitochondrial Research and Medicine, Kaohsiung Chang Gung Memorial Hospital, Kaohsiung 83301, Taiwan

**Keywords:** diabetic retinopathy, single nucleotide polymorphism, diabetes mellitus, genetics

## Abstract

Diabetic retinopathy (DR) is one of the most frequent causes of irreversible blindness, thus prevention and early detection of DR is crucial. The purpose of this study is to identify genetic determinants of DR in individuals with type 2 diabetic mellitus (T2DM). A total of 551 T2DM patients (254 with DR, 297 without DR) were included in this cross-sectional research. Thirteen T2DM-related single nucleotide polymorphisms (SNPs) were utilized for constructing genetic risk prediction model. With logistic regression analysis, genetic variations of the *FTO* (rs8050136) and *PSMD6* (rs831571) polymorphisms were independently associated with a higher risk of DR. The area under the curve (AUC) calculated on known nongenetic risk variables was 0.704. Based on the five SNPs with the highest odds ratio (OR), the combined nongenetic and genetic prediction model improved the AUC to 0.722. The discriminative accuracy of our 5-SNP combined risk prediction model increased in patients who had more severe microalbuminuria (AUC = 0.731) or poor glycemic control (AUC = 0.746). In conclusion, we found a novel association for increased risk of DR at two T2DM-associated genetic loci, *FTO* (rs8050136) and *PSMD6* (rs831571). Our predictive risk model presents new insights in DR development, which may assist in enabling timely intervention in reducing blindness in diabetic patients.

## 1. Introduction

Affecting more than one-third of patients with diabetes mellitus (DM), diabetic retinopathy (DR) is a common vision-threatening diabetic microvascular complication, which can lead to blindness [1,2]. DR is a major reason for vision loss in working-age individuals across the world, ranging from 3–7% in developing regions to 15–17% in developed countries [3,4]. Visual impairment has an impact that extends beyond the individual, as communities and economies lose earning productivity and a need for social support arises [5]. DR can advance to irreversible stages with very few symptoms. Moreover, patients with earlier stages of DR, such as mild or moderate non-proliferative diabetic retinopathy (NPDR), usually do not require treatment nor does it typically affect their vision, making them unaware of the condition [6]. Therefore, there is an essential need for screening and early detection to implement timely and effective interventions for DR.

As current approaches for treating DR are most effective when the condition is caught early, ophthalmologists need an accurate way to quickly identify patients who need treatment. Annual fundus check-ups are necessary, and depending on the degree of retinopathy, the interval between fundus examinations may need to be reduced. It is challenging to keep up with the demand to provide requisite annual screenings for the diabetic population, especially in areas with a shortage of eye-care providers [7]. Although studies highlight the potential for artificial intelligence (AI)-assisted screening algorithms in DR, there are some downsides to machine learning, including differences in camera equipment and training of screening personnel to obtain images [8,9]. All in all, screening for DR is mandatory, though it may cause a heavy burden on clinical practice for ophthalmologists if there are no adequate decision tools [7].

Predictive models have been adopted based on the risk factors identified to estimate the incidence and development of DR [10,11]. Conventional risk factors include younger age at diagnosis, longer duration of diabetes, higher systolic blood pressure (SBP) and glycosylated hemoglobin (HbA1c) levels, presence of proteinuria, dyslipidemia, and increased body-to-mass index (BMI) [12,13]. However, according to observations from the Diabetes Control and Complications Trial, HbA1C and the duration of diabetes account for just 11 percent of risk for developing DR, and other factors may account for the unexplained 89 percent risk variation [14]. These data imply that other components such as environmental and genetic determinants may contribute to the overall risk of the development and progression of DR [15,16,17].

Previous DR risk models either incorporate a multitude of laboratory variables or construct large-scale multilocus genetic risk scores [18,19,20]. However, a comprehensive model to predict DR with the combined effect of traditional risk factors and genetic variables in diabetic patients is limited [21]. Therefore, the objective of this study is to explore the key predictors and genetic variables to establish a multifactorial, practical risk prediction model for DR.

## 2. Materials and Methods

### 2.1. Study Participants

All T2DM patients aged 30 years or older who visited the endocrinology and metabolism outpatient clinics on a regular basis between February 2017 and June 2018 in Kaohsiung Chang Gung Memorial Hospital (KCGMH), Taiwan, were included in the research. Diagnosis of diabetes was based on medical records and HbA1c readings of 6.5% or higher on repeated testing, according to the American Diabetes Association Criteria [22]. A total of 606 patients were recruited, and 55 patients who were unable to complete SNP genotyping or yearly retinal photographs were excluded. All procedures adhered to the Declaration of Helsinki and the ARVO statement on human subjects. The Institutional Review Board/Ethics Committee from the Committee of Medical Ethics and Human Experiments of CGMH, Taiwan, approved of the study protocol (IRB approval no. 201601206B0C501), and all individuals gave their informed consent.

A standardized interview with each patient was conducted to collect the demographic information and medical history. The hypertension guideline established by the American College of Cardiology and the American Heart Association (ACC/AHA) in 2016 was used for blood pressure measurement [23]. Measurements were taken after the patient sat silently for 5 min with a digital automatic blood pressure device. The participant’s blood pressure was determined using the average of two measurements taken on two different occasions. The waist circumference was assessed from above the right iliac crest at the mid-axillary line, following the method in National Health and Nutrition Examination Survey (NHANES) [24]. Blood samples were collected by venipuncture for serological tests, including HbA1c, low-density lipoprotein (LDL), and high-density lipoprotein (HDL), triglycerides, and total cholesterol. Albuminuria was sorted into 3 categories based on the urine albumin-to-creatinine ratio (UACR) collected from the first voiding of urine in the morning. According to the Kidney Disease: Improving Global Outcomes (KDIGO), normoalbuminuria (A1) was defined as UACR less than 30 mg/g, microalbuminuria (A2) as UACR of between 30 and <300 mg/g, and macroalbuminuria (A3) as UACR greater than 300 mg/g [25].

DR was assessed by CR-2 digital non-mydriatic retinal camera (Canon, Tokyo, Japan). Both eyes were recorded with two retinal photographs centered on the macula. The presence of DR was determined by the agreement of fundus photo reading between two masked, trained ophthalmologists (YTH and JJL), and was assessed by a third ophthalmologist (YJC) if there was any insufficiency in the grading of the same photograph. Grading was based on the characteristic lesion present in either eye (i.e., cotton wool spots, microaneurysms, hard exudates, hemorrhages, venous beading, neovascularization, and intraretinal microvascular abnormalities), as defined by the International Clinical Diabetic Retinopathy (ICDR) classification [26]. DR severity was based on the worse eye and graded as follows: no apparent retinopathy, mild NPDR (microaneurysms only), moderate NPDR (more than only microaneurysms but less than severe NPDR), severe NPDR (more than twenty intraretinal hemorrhages in each of four quadrants, venous beading in more than two quadrants, intraretinal microvascular abnormalities in at least one quadrant), PDR (presence of neovascularization, vitreous/preretinal hemorrhage). Vision-threatening DR (VTDR) was characterized on the basis of severe NPDR, PDR, or clinically significant macular edema (CSME), as per the Eye Diseases Prevalence Research Group definition [27].

### 2.2. SNP Genotyping

The genetic material was isolated from leukocytes in peripheral blood samples. DNA was extracted with the Gentra Puregene kit (Qiagen, Venlo, The Netherlands). We selected a list of single nucleotide polymorphisms (SNPs) that have previously been linked to type 2 diabetes in an Asian/Han population. Genotyping of selected SNPs of the following genes: *CDKAL1* (rs10946398), *CDKN2A* (rs10811661), *FTO* (rs8050136), *HHEX* (rs1111875), *IGF2BP2* (rs4402960), *IRS1* (rs2943641), *KCNJ11* (rs5219), *SLC22A1* (rs622342), *TCF7L2* (rs7901695), *KCNQ1* (rs2237892), *VPS13C/C2CD4A/C2CD4B* (rs7172432), *SLC30A8* (rs13266634), *PSMD6* (rs831571) was performed with TaqMan assay, obtained from Topgen Biotechnology (Kaohsiung City, Taiwan). The following cycling conditions were used for PCR amplification: initial denaturation (95 °C for 5 min, then 30 s at 60 °C), 40 cycles of denaturation (95 °C for 3 s), annealing (60 °C for 40 s), extension (60 °C 30 s), and final extension (72 °C for 10 min). On the StepOnePlus Real Time PCR System (Thermo Fisher Scientific, Waltham, MA, USA), real-time PCR was performed using 2X AceQ Probe High ROX qPCR Master Mix (Topgen Biotechnology). Genotype-calling for allelic discrimination was executed by StepOne software v2.3 (Applied Biosystems, Grand Island, NY, USA).

### 2.3. Construction of the Combined Nongenetic and Genetic Risk Model

We constructed our risk prediction model based on previously described multiple genetic risk models [28,29]. Our nongenetic risk prediction model included the following risk factors: HbA1c, DM duration, waist circumference, systolic blood pressure, albuminuria categories, total cholesterol, and triglyceride levels. Then, to consider the greater correlation of some SNPs with DR, we added SNPs one after another to the nongenetic risk prediction model to deduce which SNPs should be included. We started by adding SNPs with the highest odds ratio in our data, to the lowest, and determined whether inserting SNPs to the prediction model could improve the area under curve (AUC) when each SNP was added. Furthermore, we also evaluated to which diabetic subgroup our risk prediction model could enhance the validity of retinopathy risk assessment by ROC curves.

### 2.4. Statistical Analysis

Quantitative variables were shown as mean ± standard deviation in descriptive analyses, while categorical data were represented as numbers and percentages. The Kolmogorov-Smirnov test was used to determine if the parameters had a Gaussian distribution. Mann-Whitney U test was employed to compare continuous variables, while Fisher’s exact or Pearson’s chi-squared (χ2) tests were used to analyze categorical data as appropriate. The association between the 13 SNPs and the risk of DR was explored using additive, dominant, and recessive models. The difference in genotype and allele frequencies, and the interaction between 13 SNPs with DR risk were analyzed using multivariate logistic regression adjusted for waist circumference, SBP, DM duration, HbA1c, albuminuria categories, total cholesterol, and triglycerides. Odds ratios (ORs) with 95% confidence intervals (CIs) were obtained for independently associated genetic variants. To better investigate the significant differences between DR and genetic variants in DR and non-DR groups, multivariable logistic regression analyses further adjusting for DM medication usage was performed. We conducted a post hoc power analysis to calculate the statistical power using the G*Power 3.1.9 (from Heinrich-Heine-Universität Düsseldorf, Franz Faul, Universitat Kiel, Kiel, Germany), assuming a 2-tailed test at 5% alpha level.

To evaluate the discriminating ability of our risk prediction model for DR patients, we calculated the area under the receiver-operating characteristic (ROC) curve. According to the method of Hanley and McNeil [30], we compared the AUCs of different nongenetic and genetic risk prediction models. All statistical analyses were performed using SPSS 20.0 (SPSS Inc., Chicago, IL, USA). A *p* value of <0.05 was considered as significant.

## 3. Results

### 3.1. Subject Characteristics

In our study, a total of 551 participants with T2DM were included in the analysis, with 254 (46%) patients being diagnosed with DR. Fifty-five subjects were excluded due to insufficient SNP genotyping data or fundus photographs. No significant differences were found in the characteristics of either the included or excluded group of participants except for waist circumference (90.92 ± 0.46 cm vs. 94.72 ± 1.58 cm, *p* = 0.01). The comparisons of baseline demographic data between the DR and non-DR group are shown in Table 1. Participants with DR were older, female, and had larger waist circumferences overall. The DR group had significantly higher levels of SBP (*p* = 0.002) and HbA1c (*p* < 0.001), longer duration of DM (*p* < 0.001), and worse albuminuria (*p* = 0.02). The difference of lipid profiles between the two groups was insignificant.

### 3.2. Association between SNPs and DR Risk

**Figure 1:**
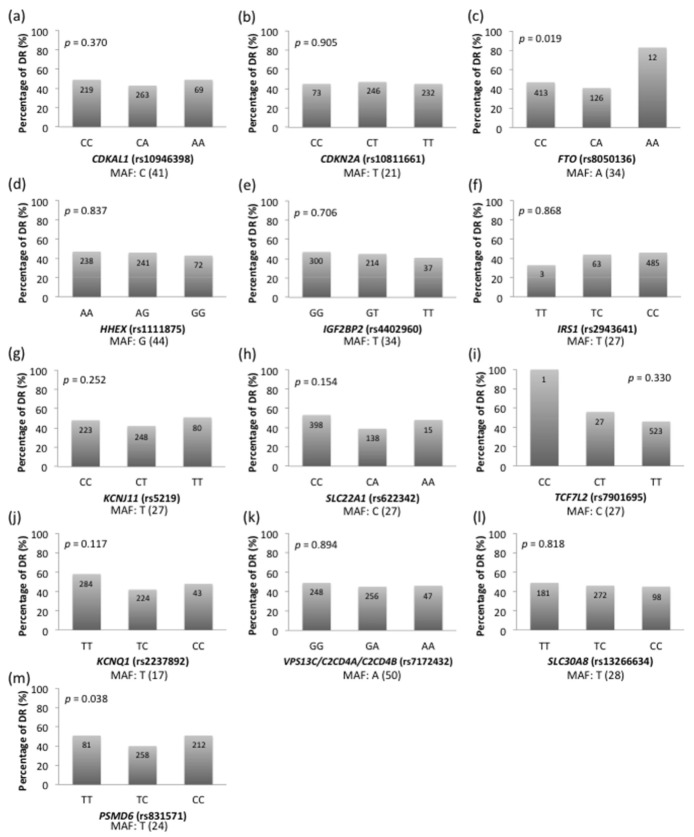
Comparison of diabetic retinopathy (DR) prevalence in type 2 diabetes mellitus (T2DM) patients according to 13 single nucleotide polymorphisms (SNPs) previously found to be associated with type 2 diabetes in Asian populations. (**a**–**m**) The number in the bar represents the total number of subjects in the genotype. The global minor allele frequency (MAF) of each SNP is presented at the bottom of each subfigure with percentage in brackets.

The association of the polymorphisms with DR are shown in Figure 1. The DR prevalence increased significantly in cases with the risk allele (A) of *FTO* (rs8050136). The percentage of DR was 46.5%, 41.3%, and 83.3% in patients with CC, CA, and AA genotype, respectively (*p* = 0.019). Furthermore, patients with the TC genotype of *PSMD6* (rs831571) had a lower DR prevalence (40.3%) than those with the TT (50.6%) and CC genotypes (51.4%). The DR prevalence of patients with genetic variants of *PSMD6* (rs831571) was significantly different (*p* = 0.038).

Backward stepwise logistic regression analysis of the 13 SNPs using three genetic models (additive, dominant, and recessive models) showed that *FTO* (rs8050136) and *PSMD6* (rs831571) were independently associated with DR in the additive model (FDR-corrected *p* = 0.092 and FDR-corrected *p* = 0.044, respectively; Table 2) and recessive model (FDR-corrected *p* = 0.071 and FDR-corrected *p* = 0.044, respectively; Table 3), further suggesting that the A allele in *FTO* (rs8050136) and C allele in *PSMD6* (rs831571) were risk alleles for DR. Results from logistic regression models demonstrated that SBP (OR, 1.012; 95% CI, 1.002–1.022; *p* = 0.03), DM duration (OR, 1.067; 95% CI, 1.040–1.095; *p* < 0.001), HbA1c (OR, 1.288; 95% CI, 1.217–1.812; *p* < 0.001), and albuminuria (OR, 1.536; 95% CI, 0.971–1.709; *p* = 0.079) were independently associated with DR; whereas waist circumference, total cholesterol, and triglycerides were not. After adjustment for variables including waist circumference, SBP, DM duration, HbA1c, albuminuria categories, lipid profiles, and for DM medications, patients with *FTO* (rs8050136) polymorphism were significantly associated with increased DR risk (OR, 5.851; CI, 1.201–28.510; FDR-corrected *p* = 0.039), whereas those with *PSMD6* (rs831571) polymorphisms were not (*p* = 0.076) (Table 4). The post-hoc power analysis was performed for the association of each SNP grouping in Table 2, Table 3 and Table 4. The results demonstrated that the power analyses were between 0.176 and 0.359 for *FTO* (rs8050136), and between 0.09 and 0.659 for *PSMD6* (rs831571) in the additive model (Table 2). The power analyses were 0.657 and 0.716 for *FTO* (rs8050136) variant, and 0.647 and 0.472 for the *PSMD6* (rs831571) variant in recessive models (Table 3) and when adjusted for DM medication usage (Table 4), respectively.

**Figure 2:**
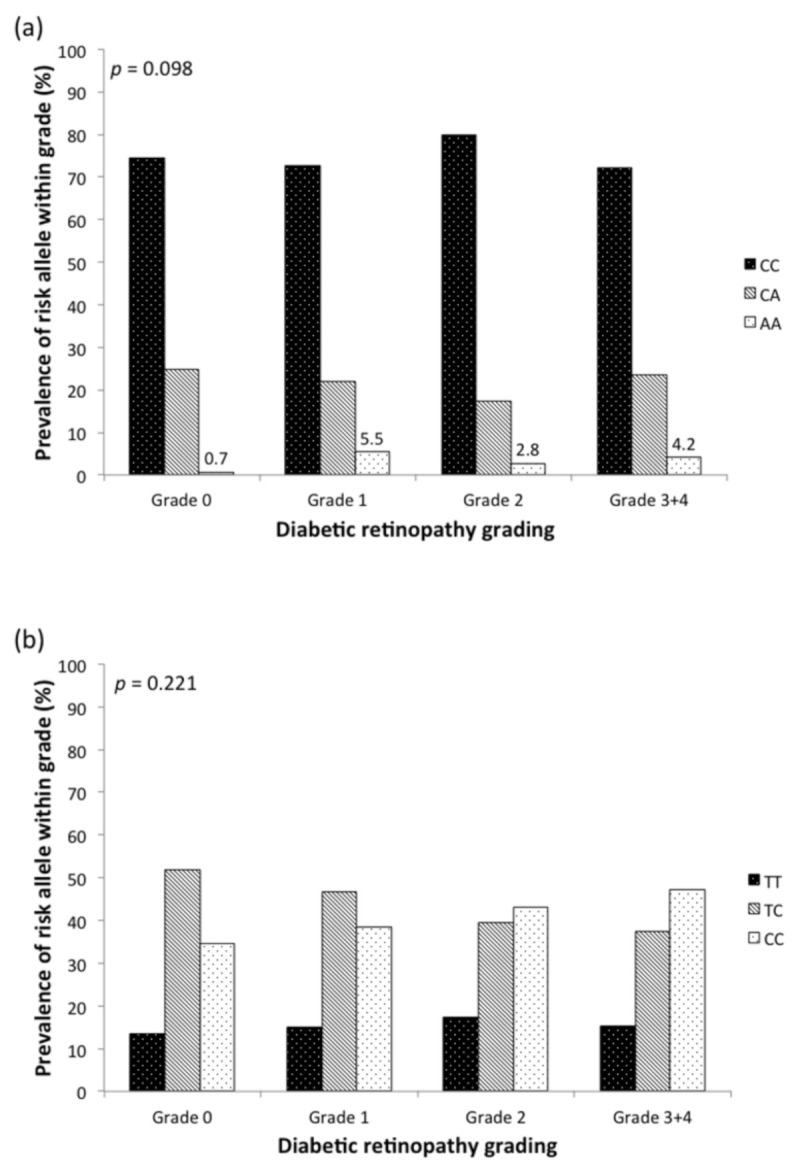
Comparison of the prevalence of risk alleles in different DR stages in T2DM patients (**a**) *FTO* rs8050136 (**b**) *PSMD6* rs831571. DR grading as follows: Grade 0, no DR; grade 1, mild NPDR; grade 2, moderate NPDR; grade 3, severe NPDR; grade 4, PDR. *p* value from chi-square test.

**Figure 3:**
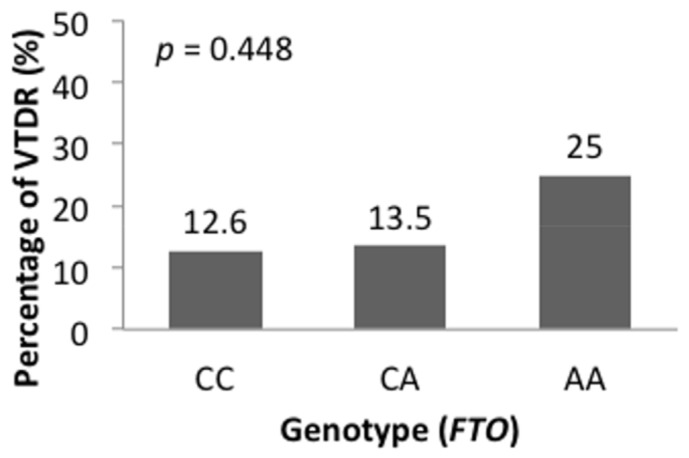
The prevalence of vision-threatening DR (including severe NDPR and PDR) in T2DM patients with genotypic variants (CC, CA, AA) of *FTO* rs8050136. *p* value from chi-square test.

**Figure 4:**
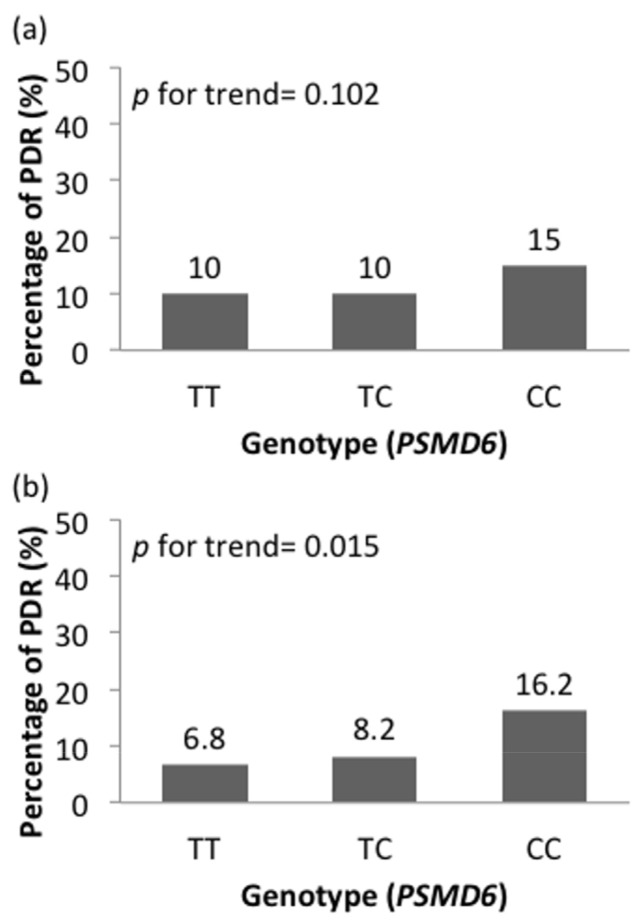
The effect of thiazolidinediones (TZD) usage on the prevalence of proliferative diabetic retinopathy (PDR) in T2DM patients of *PSMD6* rs831571 variant. The bar chart indicates the PDR prevalence in patients (**a**) without using and (**b**) using TZD according to genotype of *PSMD6*.

The associations of risk allele frequency with DR severity are shown in Figure 2. Both *FTO* (rs8050136) and *PSMD6* (rs831571) demonstrated a trend of higher DR severity levels in subjects with risk alleles. Figure 3 shows that the prevalence of VTDR was higher in patients carrying more risk alleles of *FTO* (rs8050136) at 12.6%, 13.5%, and 25.0%, respectively (*p* = 0.448). For *PSMD6* (rs831571), patients carrying more risk alleles had a higher trend of PDR prevalence (Figure 4a). Carriers of the C allele showed a significant increasing trend of PDR prevalence in patients without using thiazolidinedones (*p* = 0.015) (Figure 4b).

We also investigated the association of *FTO* (rs8050136) with obesity. Our results indicated a trend (*p* = 0.087) between DR and waist circumference under per-allele comparison of rs8050136, despite being insignificant.

### 3.3. Validation of Risk Prediction Model

**Figure 5:**
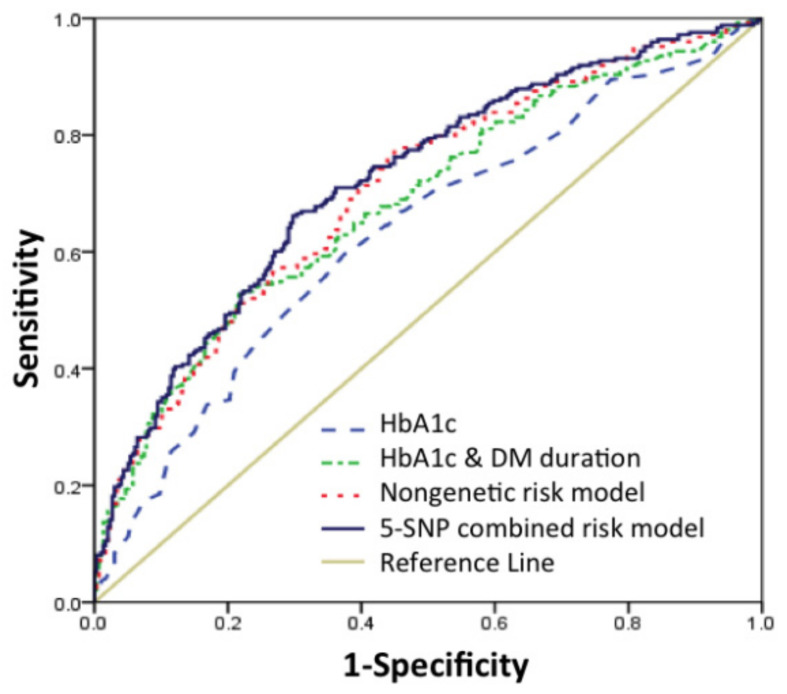
Receiver operating characteristic (ROC) curves and area under the curve (AUC) in diabetic patients. Blue line: HbA1c risk score (AUC = 0.631); green line: HbA1c and DM duration risk model (AUC = 0.685); red line: nongenetic risk model (AUC = 0.704); navy line: 5-SNP combined risk model (AUC = 0.722). The beige line represents the reference line (no discrimination).

To verify the discriminatory accuracy of our genetic risk prediction model with conventional DR risk factors, we evaluated the different AUC estimates in nongenetic, genetic, and combined risk prediction models. The nongenetic risk prediction model was determined from known nongenetic risk factors for DR, including HbA1c, DM duration, waist circumference, SBP, albuminuria categories, dyslipidemia et al. The AUC for HbA1c was 0.631 (95% CI, 0.584–0.678), which increased to 0.685 (95% CI, 0.640–0.730) after including DM duration. The AUC for all nongenetic risk factors (waist circumference, SBP, DM duration, HbA1c, albuminuria categories, cholesterol, triglycerides) was 0.704 (95% CI, 0.661–0.748) (Figure 5).

**Figure 6:**
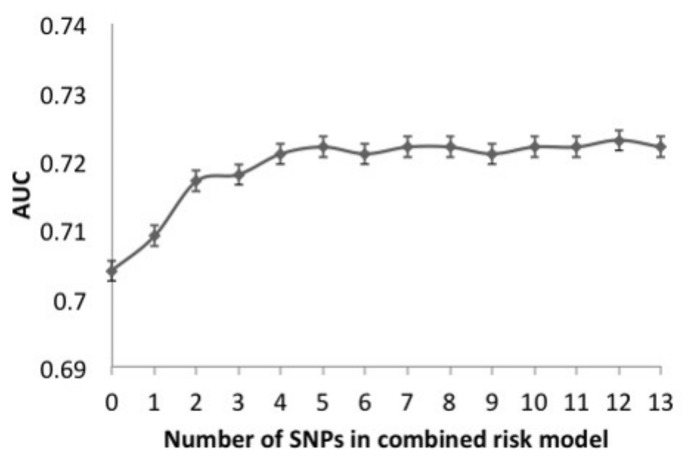
Area under the ROC of nongenetic and genetic combined risk model based on increasing number of SNPs. SNPs were added in order of the odds ratio (see Table 2); starting with *FTO* based on 1 SNP and ending with *SLC22A1* (13 SNPs). Nongenetic risk model (0 SNP) was based on SBP, DM duration, waist circumference, HbA1c, albuminuria categories, total cholesterol, high-density lipoprotein, low-density lipoprotein, triglycerides. ROC, receiver operating characteristic curve; AUC, area under ROC curve.

We added SNPs to the nongenetic risk prediction model on an individual basis, in order of the SNPs with the greatest OR in logistic regression analysis (Table S1), in order to build a combined risk prediction model using the most practical method. Subsequently, we evaluated the AUC after adding each SNP (Figure 6). The discriminative accuracy of the predictive model went up with inclusion of each SNP, up to a total 5 SNPs. These SNPs were *FTO* (rs8050136), *PSMD6* (rs831571), *TCF7L2* (rs7901695), *KCNJ11* (rs5219), *KCNQ1* (rs2237892) in order of addition. This 5-SNP combined risk prediction model had an AUC of 0.722 (95% CI, 0.679–0.764) (Figure 5).

**Figure 7:**
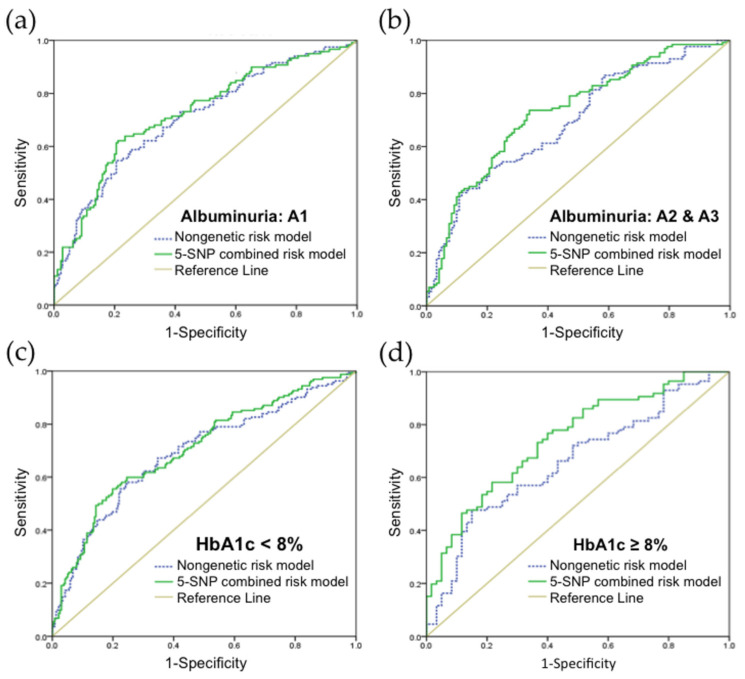
Receiver operating characteristic (ROC) curves and area under the curve (AUC) in different diabetic subgroups; (**a**) diabetic patients in albuminuria category A1. Blue line: nongenetic risk model (AUC = 0.708); green line: 5-SNP combined risk model (AUC = 0.724); (**b**) diabetic patients in albuminuria category A2 and A3. Blue line: nongenetic risk model (AUC = 0.693); green line: 5-SNP combined risk model (AUC = 0.731); (**c**) diabetic patients with HbA1c levels <8%. Blue line: nongenetic risk model (AUC = 0.690); green line: 5-SNP combined risk model (AUC = 0.707); (**d**) diabetic patients with HbA1c levels ≥8%. Blue line: nongenetic risk model (AUC = 0.659); green line: 5-SNP combined risk model (AUC = 0.746). The beige line represents the reference line (no discrimination).

In addition, we assessed the discriminatory ability of our model in different subgroups. After classifying the DR subjects into UACR < 30 mg/g (albuminuria category A1, *n* = 295) and UACR ≥ 30 mg/g (albuminuria categories A2 and A3, *n* = 256) (Figure 7a,b), the combined nongenetic and genetic risk prediction model (AUC = 0.731; 95% CI, 0.669–0.793) showed a larger increase in discrimination ability than the conventional nongenetic risk prediction model (AUC = 0.693; 95% CI, 0.628–0.757) in the group with albuminuria categories A2 and A3 than that in A1 group. Moreover, in diabetic patients with well glycemic control (HbA1c < 8%, *n* = 402) and poor glycemic control (HbA1c ≥ 8%, *n* = 149) (Figure 7c,d) [31], we found that the discrimination ability increased to a greater extent in subjects with poor glycemic control when comparing between the 5-SNP combined risk prediction model (AUC = 0.746; 95% CI, 0.666–0.825) and nongenetic risk prediction model (AUC= 0.659; 95% CI, 0.570–0.748) alone.

## 4. Discussion

In this study, we presented novel genetic T2DM related genetic variants associated with DR risk and progression and established a comprehensive multifactorial prediction model. Of the 13 T2DM genetic loci, we found *FTO* rs8050136 and *PSMD6* rs831571 as genetic markers of DR development, independent of traditional risk factors. In our analysis, a risk prediction model based on the 5 SNPs with the greatest OR appeared to distinguish patients with and without DR similarly, as well as the initial genetic risk model based on 13 SNPs. Our findings may provide a practical method for screening T2DM patients who are susceptible of DR, and a promising contribution to the understanding of the mechanisms underlying DR progression.

Diabetes is known for its microvascular and macrovascular complications that contribute to long-term damage and failure of organ systems. Microangiopathy is associated with increased damage in different organs, thus promoting retinopathy, nephropathy, and neuropathy. Oxidative stress and inflammation on vascular alterations were found to affect DR development [32,33]. Obesity plays an important role in microvascular dysfunction as well [34]. Previous studies have indicated the importance of obesity-related factors and oxidative stress in DR risk. Taken together, these theories imply that microvascular damage and metabolic physiology in hyperglycemia may form a vicious cycle with multiorgan consequences. Given the high morbidity and mortality associated with T2D complications, identifying genetic risk factors for T2D complications is critical.

We found the DR risk increased significantly in patients with the *FTO* (rs8050136) genetic polymorphism, even after adjustment for traditional risk factors and different DM medications use. Subjects with risk allele (A) of this variant showed a trend for DR progression. In both Western and Chinese populations, the *FTO* polymorphism rs8050136 has been linked to obesity markers (BMI, waist, and hip circumference) and glucose homeostasis [35,36,37]. Bravard et al. demonstrated that *FTO* mRNA and protein levels in skeletal muscle from T2DM patients increased, implying that FTO may have a role in oxidative metabolism [38]. Interestingly, variants in the *FTO* gene correlated to a decrease in the presence of obesity [39], which is a risk factor for retinopathy [40]. Our subgroup analysis implied that rs8050136 risk allele (A) and obesity may be correlated. Although the number of risk alleles in rs8050136 was related to increased waist circumference, the results were insignificant possibly due to the limited genotype population. Therefore, the exact mechanism underlying the FTO function and DR is still illusive and demands further research.

The encoded protein of the *PSMD6* gene is a member of the ubiquitin-proteasome system and a subunit of the 26S proteasome (UPS) [41]. The UPS is the foundation of regulating cell cycle, protein quality control, and differentiation and numerous signal transduction pathways. In previous GWAS studies, the *PSMD6* rs831571 variation was discovered to be a T2DM susceptibility locus [42,43]. In the retina and kidney, Aghdam and Sheibaani have expressed that hyperglycemia and oxidative stress modulate UPS activity and altered UPS activity in diabetes may give rise to the formation of retinal microvascular complications [44]. The *PSMD6* variation rs831571 was shown to be strongly linked to the development of DR in our study, but became insignificant after adjustment for DM medication use. Previously, in a Chinese cohort, the *PSMD6* variant rs831571 was observed to be closely related to the therapeutic properties of rosiglitazone. It was inferred that as the 26S proteasome is a subunit of UPS, SNPs in the *PSMD6* gene may alter the oral antidiabetic drug effects due to its impact on insulin secretion and sensitivity [45]. Nevertheless, the association between PSMD6 and rosiglitazone use cannot explain the effect of different genotype of *PSMD6* on DR in our cohort. After excluding thiazolidinedione users, we discovered the risk allele (C) of *PSMD6* (rs831571) to be correlated with DR severity progression, more specifically, the advancement into PDR.

The development of DR may be related to high levels of inflammation and insulin resistance in diabetic individuals with abdominal obesity [46,47,48]. Besides *FTO* polymorphisms, *KCNJ11* rs5219, *KCNQ1* rs2237892, and *TCF7L2* rs7901695 variations have previously been linked to obesity in patients with T2DM. In Caucasians and East Asian groups, the *KCNJ11* rs5219 polymorphism has been identified as a risk factor for developing T2DM. It can impact the insulin secretion pathway by decreasing ATP sensitivity of the KATP channel, resulting in suppression of insulin secretion. This variant also plays a major role in blood pressure and HbA1c levels in diabetes [49]. By impairing β-cell function, KCNQ1 confers a risk for T2DM, and was realized to be strongly linked to a greater likelihood of T2DM in European and East Asian populations [50,51]. In terms of diabetes complications, it was suggested that the *KCNQ1* rs2237892 variant may contribute to susceptibility in macrovascular disease, diabetic nephropathy, and diabetic retinopathy [52,53]. In previous studies, there was a significant association of *KCNQ1* rs2237892 and *TCF7L2* rs7901695 variants with impaired lipid parameters [54,55]. Although hyperglycemia is the main initiator in DR, dyslipidemia is also associated with the development of this disease [56]. In an animal experimental model, obesity was found to alter hyperglycemia-induced epigenetic modifications, accelerating mitochondrial damage and DR [57]. Despite the fact that the associations of *KCNJ11* rs5219, *KCNQ1* rs2237892, and *TCF7L2* rs7901695 with DR are not significant in our data, we believe in a more long-term longitudinal study they may still influence the development and progression of DR according to our predictive model.

We established a risk prediction model based on the SNPs that were most consistently linked to T2DM and discovered a “dose-response” relationship between the risk prediction model and DR—to an extent, the more SNPs involved, the greater the risk of DR. The risk prediction model based on the five SNPs with the highest ORs in our data appeared to discriminate between patients with and without DR equally effectively as the initial model constructed on the selected 13 SNPs. The AUC increased to 0.722 by combining the 5-SNP genetic risk prediction model with the nongenetic risk prediction model, demonstrating good diagnostic accuracy. As the capacity of outpatient clinic follow-up may be inadequate, precise discrimination may help to establish a personalized schedule for fundus follow-up to fulfill the difference between those at greater and lower risk of developing diabetic retinopathy. We investigated in-depth the extent to which risk prediction model can enhance the accuracy of ROC curves for retinopathy risk assessment. Regarding diabetic patients with albuminuria, the 5-SNP genetic risk prediction model (AUC = 0.731) performed better than conventional prediction model (AUC = 0.693) alone. In subgroup analysis on the degree of glycemic control, the proportion of discriminative accuracy increased with a larger amount in persons with poor glycemic control. In brief, our 5-SNP prediction model may have greater potential clinical value in certain diabetic populations. In patients with diabetic nephropathy (UACR ≥ 30 mg/g) or insufficient glycemic control (HbA1c levels ≥ 8%), shortened intervals between fundus examinations and intensive diabetes self-management education should be arranged.

The strengths of this study included a comprehensive systemic and medication history assessment, with standardized assessment of DR based on retinal photographs. We also recognize some limitations in our study. Our study population only consisted of Asian ethnicities and the patient number was relatively limited, thus further validation of the risk prediction model in other populations of different ethnicities may be needed. Although the excluded population revealed significance in wider waist circumference and were older when compared to our included subject population, we believe the features may be associated. Elderly diabetic patients are more likely to have a longer duration of DM and wider waist circumferences and reduced ability in completing all aspects required in the study. Lastly, due to this being a cross-sectional study, a long-term longitudinal study may be necessary on further identification of SNP genotypes in terms of DR progression.

## 5. Conclusions

Identification of persons at risk of DR development and progression is of paramount importance in diabetic populations. Our study provides evidence of a novel association between *FTO* rs8050136 and *PSMD6* rs831571 polymorphisms with DR development. The predictive value of our combined nongenetic and genetic risk prediction model may aid in better identifying a subset of patients at greater risk of DR in clinical practice.

## Figures and Tables

**Table 1:** Baseline characteristics of patients.

	Non-DR	DR	*p*-Value
*n*	297	254	
Age (year)	60.78 ± 9.81	61.26 ± 9.71	0.648 ^a^
Male sex, no. (%)	155 (53.4)	142 (54.4)	0.822 ^b^
Waist circumference (cm)	90.30 ± 10.38	91.64 ± 11.29	0.143 ^a^
SBP (mmHg)	136.56 ± 18.80	140.90 ± 19.86	0.002 ^a^
DBP (mmHg)	77.53 ± 11.89	78.41 ± 12.49	0.375 ^a^
Duration of DM (year)	14.70 ± 6.47	19.15 ± 8.73	<0.001 ^a^
HbA1c (%)	7.24 ± 0.88	7.72 ± 1.14	<0.001 ^a^
Total cholesterol (mg/dL)	168.98 ± 1.75	167.27 ± 2.07	0.305 ^a^
HDL (mg/dL)	47.42 ± 0.70	46.89 ± 0.89	0.090 ^a^
LDL (mg/dL)	89.94 ± 1.32	90.18 ± 1.58	0.601 ^a^
Triglycerides (mg/dL)	139.92 ± 5.26	134.51 ± 4.81	0.678 ^a^
Albuminuria categories ^c^			0.02 ^b^
A1, no. (%)	176 (59.3)	121 (47.6)	
A2, no. (%)	102 (34.3)	101 (39.8)	
A3, no. (%)	19 (6.4)	32 (12.6)	

^a^ Mann Whitney U test; ^b^ Chi-square test; ^c^ data from Kidney Disease: Improving Global Outcomes (KDIGO) 2012 Clinical Practice Guideline. DR: diabetic retinopathy; SBP: systolic blood pressure; DBP: diastolic blood pressure; DM: diabetes mellitus; HDL: high-density lipoprotein; LDL: low-density lipoprotein.

**Table 2:** Backward stepwise regression analysis on the association between SNPs and diabetic retinopathy using the additive model.

Genetic Variables	Genotypic Value			1 vs. 0			2 vs. 1			2 vs. 0		
	0	1	2	S.E.	OR (95% CI)	*p*-Value	S.E.	OR (95% CI)	*p*-Value	S.E.	OR (95% CI)	*p*-Value
*FTO* (rs8050136)	CC	CA	AA	1.120	0.789 (0.509, 1.223)	0.290	0.829	5.650 (1.111, 28.571)	0.044	3.376	4.451 (0.905, 21.888)	0.095
*PSMD6* (rs831571)	TT	TC	CC	2.125	0.666 (0.386, 1.150)	0.174	0.204	1.692 (1.135, 2.525)	0.020	0.177	1.127 (0.645, 1.972)	0.674

Genetic variables were adjusted for waist circumference, SBP, duration of DM, albuminuria categories, HbA1c, total cholesterol, triglycerides as covariates. *p* values were false discovery rate (FDR) corrected. Post-hoc power analysis of *FTO* (rs8050136): 1 vs. 0, power = 0.176; 2 vs. 1, power = 0.332; 2 vs. 0, power = 0.359. Post-hoc power analysis of *PSMD6* (rs831571): 1 vs. 0, power = 0.511; 2 vs. 1, power = 0.659; 2 vs. 0, power = 0.09. S.E.: standard error; OR: odds ratio; CI: confidence interval; vs.: versus.

**Table 3:** Backward stepwise regression analysis on the association between SNPs and diabetic retinopathy using the recessive model.

Genetic Variables	Alleles	S.E.	OR (95% CI)	*p*-Value
*FTO* (rs8050136)	CC + CA vs. AA	3.569	4.605 (0.944, 22.454)	0.071
*PSMD6* (rs831571)	TT + TC vs. CC	4.762	1.519 (1.044, 2.212)	0.044

Genetic variables were adjusted for waist circumference, SBP, duration of DM, albuminuria categories, HbA1c, total cholesterol, and triglycerides as covariates. *p* values were false discovery rate (FDR) corrected. Post-hoc power analysis: *FTO* (rs8050136), power = 0.657; *PSMD6* (rs831571), power = 0.647. S.E.: standard error; OR: odds ratio; CI: confidence interval.

**Table 4:** Backward stepwise regression analysis on the association between SNPs rs8050136 and rs831571 on diabetic retinopathy risk using the recessive model, adjusted for DM medication usage.

Variables	S.E.	OR (95% CI)	*p*-Value
DM medications			
Insulin	0.262	2.609 (1.561, 4.360)	0.003
GLP-1 receptor agonists	0.499	2.838 (1.067, 7.553)	0.043
genetics			
*FTO* (rs8050136)	4.780	5.851 (1.201, 28.510)	0.041
*PSMD6* (rs831571)	3.144	1.414 (0.964, 2.074)	0.076

Genetic variables adjusted for waist circumference, systolic blood pressure (SBP), duration of diabetes mellitus (DM), albuminuria categories, HbA1c, total cholesterol, triglycerides, DPP-4 inhibitors, sulfonylureas, glinides, SGLT-2 inhibitors, α-glucosidase inhibitors, biguanides, thiazolidinediones, insulin, GLP-1 receptor agonists. *p* values were false discovery rate (FDR) corrected. Post-hoc power analysis: *FTO* (rs8050136), power = 0.716; *PSMD6* (rs831571), power = 0.472. S.E.: standard error; OR: odds ratio; CI: confidence interval.

## Data Availability

Data is fully available upon reasonable request to corresponding author.

## Supplementary Materials

The following are available online at https://www.mdpi.com/article/10.3390/jpm11080689/s1, Table S1: Multiple analysis of SNPs on DR risk with logistic regression using enter method.
