# Supplementary material for: Multiple Single Nucleotide Polymorphism Testing Improves the Prediction of Diabetic Retinopathy Risk with Type 2 Diabetes Mellitus
Source: J Pers Med. 2021 Jul 21;11(8):689. doi: 10.3390/jpm11080689 (PMC8398882; doi:10.3390/jpm11080689)
Supplement: Supplementary file 1 [file jpm-11-00689-s001.zip › jpm-1284872-supplementary.pdf]

Table S1. Multiple analysis of SNPs on DR risk with logistic regression using enter method

		OR (95% CI)
CDKAL1 (rs10946398)	CC+CA vs. AA	1.237 (0.711, 2.153)
CDKN2A (rs10811661)	CC+CT vs. TT	0.948 (0.649, 1.384)
FTO (rs8050136)	CC+CA vs. AA	5.617 (1.090, 28.950)
HHEX (rs1111875)	AA+AG vs. GG	0.956 (0.542, 1.689)
IGF2BP2 (rs4402960)	GG+GT vs. TT	0.867 (0.409, 1.836)
IRS1 (rs2943641)	TT+TC vs. CC	0.900 (0.504, 1.607)
KCNJ11 (rs5219)	CC+CT vs. TT	1.515 (0.898, 2.558)
SLC22A1 (rs622342)	CC+CA vs. AA	1.706 (0.566, 5.142)
TCF7L2 (7901695)	CC+CT vs. TT	1.491 (0.641, 3.467)
KCNQ1 (rs2237892)	TT+TC vs. CC	1.145 (0.787, 1.665)
VPS13C/C2CD4A/C2CD4B (rs7172432)	GG+GA vs. AA	0.931 (0.639, 1.357)
SLC30A8 (rs1326634)	TT+TC vs. CC	0.867 (0.580, 1.297)
PSMD6 (rs831571)	TT+TC vs. CC	1.562 (1.064, 2.294)

Genetic variables adjusted for waist circumference, SBP, duration of DM, albuminuria categories, HbA1c, total cholesterol, high-density lipoprotein, low-density lipoprotein, triglycerides. OR: odds ratio; CI: confidence interval; vs.: versus.
